# Breast incidentaloma: Cardiac PET readers beware

**DOI:** 10.1016/j.radcr.2024.10.152

**Published:** 2024-12-02

**Authors:** Nirali Munshi, Julio Perez-Downes, Amie Leon, Haley Letter

**Affiliations:** a Edward Via College of Osteopathic Medicine, 2265 Kraft Dr, Blacksburg, VA, 24060, USA; b Mayo Clinic Florida, Department of Cardiovascular Medicine, 4500 San Pablo Rd, Jacksonville, FL, 32224, USA; c Mayo Clinic Florida, Department of Radiology, Division of Breast Imaging, 4500 San Pablo Rd, Jacksonville, FL, 32224, USA

**Keywords:** Breast cancer, Ductal carcinoma in situ, Atypical ductal hyperplasia, Positron emission tomography, Computed tomography

## Abstract

Breast cancer remains one of the most common causes of cancer and cancer-related death in women. With increases in medical imaging utilization, incidentally detected cancer has become more prevalent. Specifically, breast cancer can be incidentally detected on nuclear cardiac imaging scans due to its high metabolic activity and because the tumor may fall within the field of view during these studies. We report a unique case of ductal carcinoma in situ found on Nitrogen-13 ammonia myocardial perfusion positron emission tomography-computed tomography (PET-CT) in a patient undergoing work up for chest pain.

## Introduction

Breast cancer is the most common cause of cancer in women and the leading cause of cancer-related mortality [1]. Breast cancer diagnosis occurs through either routine screening, or investigation of a presenting symptom, such as pain or a palpable mass [2]. Half of all cases occur in women who have no risk factors other than age and sex, highlighting the importance of routine screening [3]. While mammographic screening is the only modality proven to reduce mortality from breast cancer, incidental detection of breast cancer can be found on other imaging studies, particularly those that routinely include the breasts in the field of view, such as cardiac imaging. Myocardial perfusion imaging with technetium-99m (Tc-99m) sestamibi, a common imaging technique utilized for assessment of myocardial ischemia, is one of the imaging studies in which the breast is included in the field of view, and one in which many cases of breast cancer have been incidentally detected. Our case highlights the importance of comprehensive evaluation of cardiac imaging, as it illustrates the case of a patient in whom an incidental focus of radiotracer uptake was noted in the right breast during a Nitrogen-13 (N-13) ammonia myocardial perfusion imaging positron emission tomography- computed tomography (PET-CT) performed for ischemia assessment.

## Case report

A 78-year-old female initially presented to the emergency room with shortness of breast and chest pain. Her past medical history was significant for mitral prolapse without intervention, spinal stenosis, and COVID-10 pneumonia one year prior. The chest pain was substernal in location, occurred at rest and stress, without exacerbating or alleviating factors. Her EKG upon presentation was without acute ST segment changes concerning for ischemia. Cardiac troponins were negative. Chest CT was performed and was negative for pneumonia, pulmonary embolus, or effusion. Laboratory data was significant only for hypokalemia of 2.9 millimol/L (normal range 3.7-5.2 millimol/L), which was repleted with intravenous potassium during admission. Cardiology was consulted and recommended a cardiac CT angiogram which revealed no coronary calcium (calcium score was 0) and no coronary stenosis. Given the patient's symptoms of chest pain, microvascular coronary artery disease was the main differential diagnosis consideration. The patient was discharged in stable condition with plan for outpatient nuclear cardiac imaging.

**Fig. 1:**
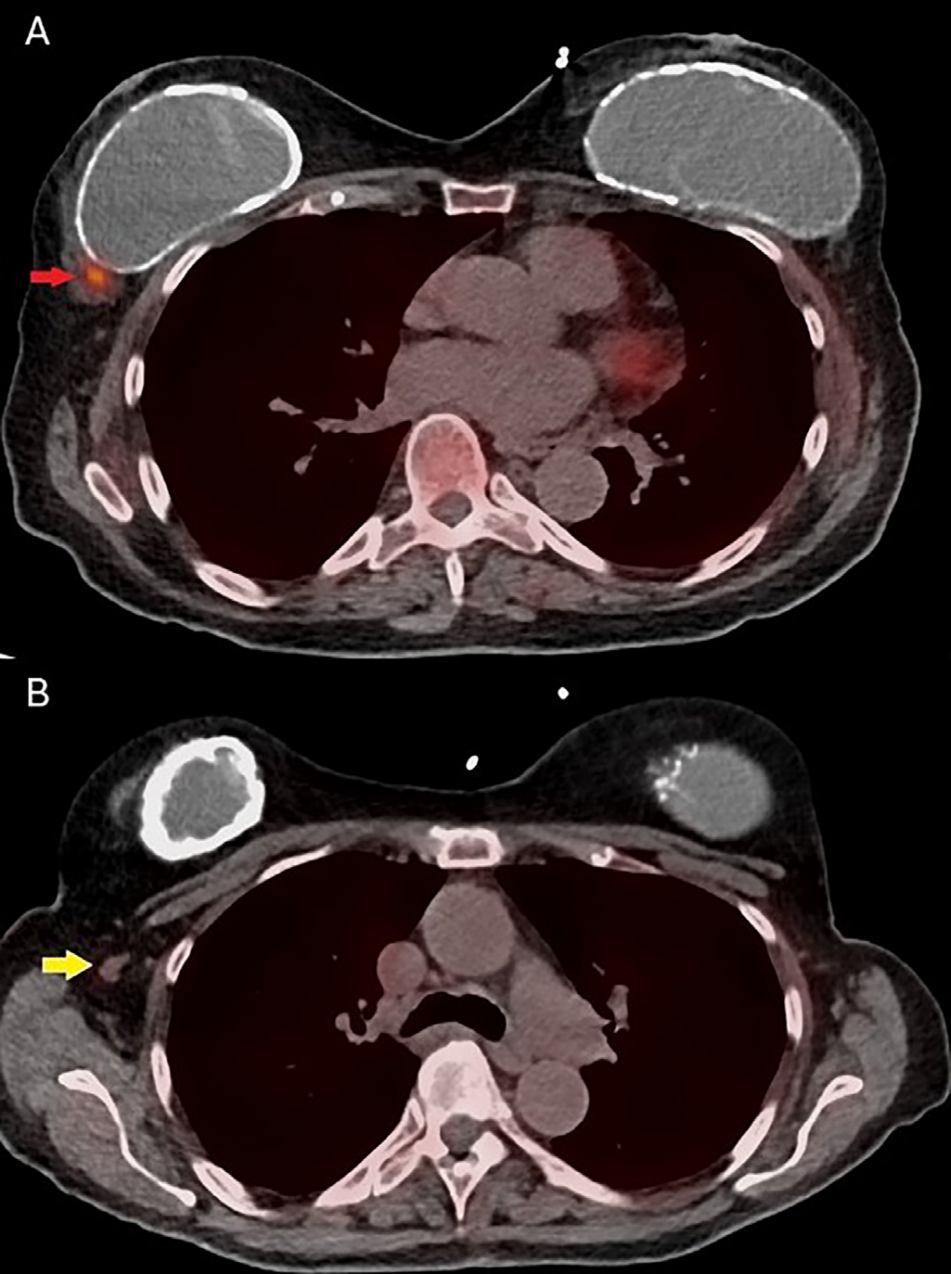
(A) Axial PET-CT image demonstrates uptake in the outer right breast along the implant capsule (red arrow). (B) There is mild uptake within a right axillary lymph node (yellow arrow).

**Fig. 2:**
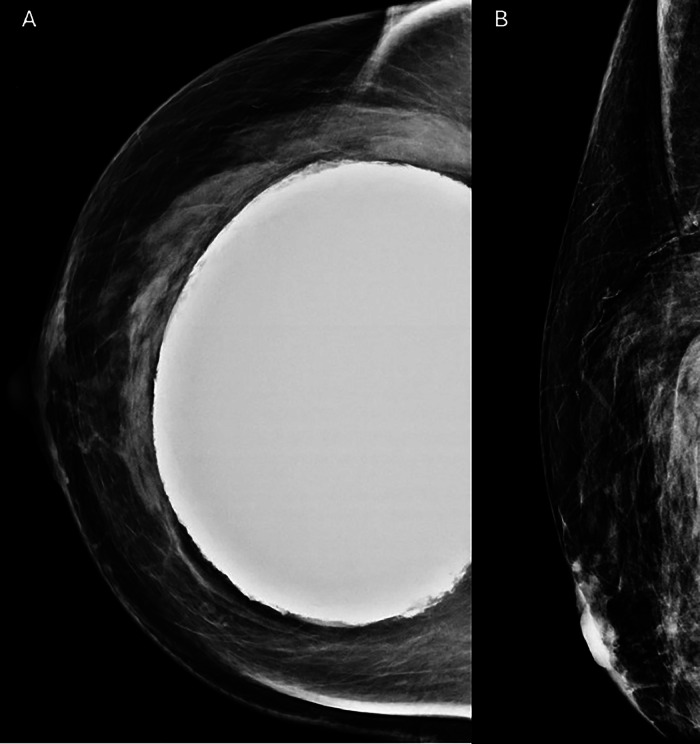
Craniocaudal view of the right breast (A) and exaggerated craniocaudal implant displaced view (B) of the right breast demonstrate a silicone implant without mammographic correlate for the area of uptake in the outer breast seen on PET.

**Fig. 3:**
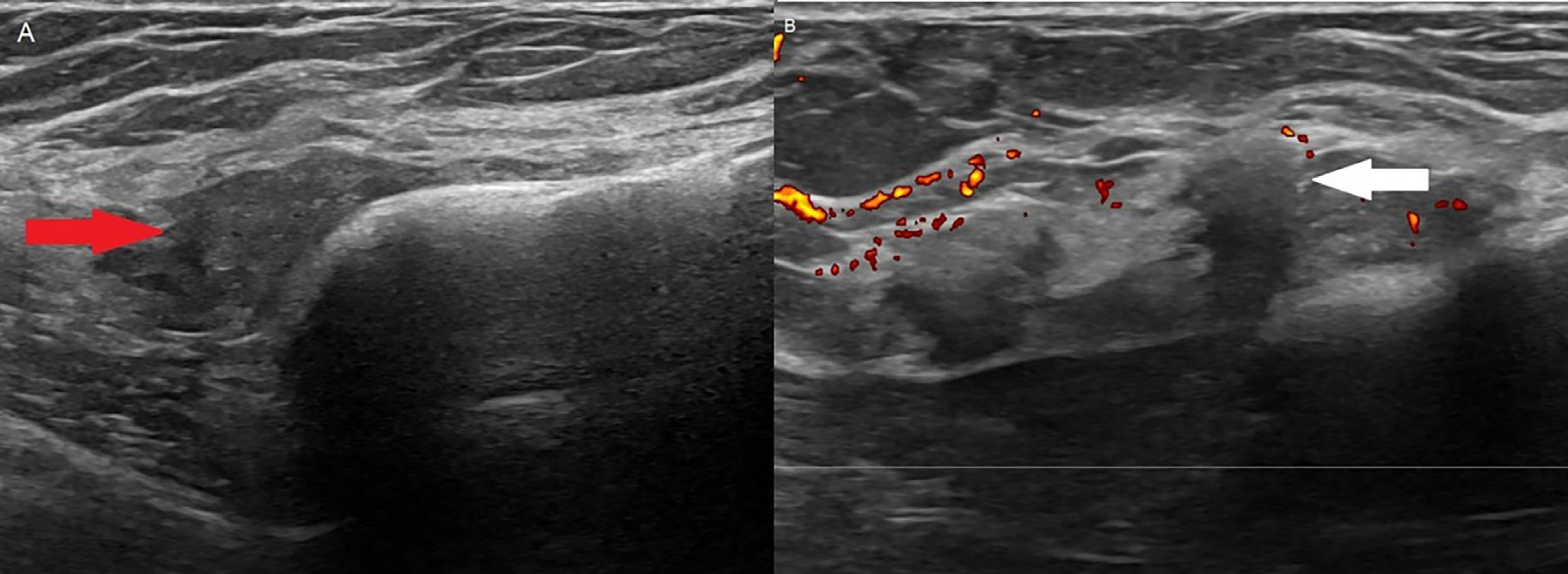
Grayscale ultrasound image (A) demonstrates an irregular, hypoechoic mass (red arrow) measuring approximately 2.2×0.8×1.9 cm along the implant capsule. Power doppler image (B) demonstrates the mass (white arrow) with no significant vascularity.

Within 2 weeks of discharge from the hospital, the patient underwent (N)-13 ammonia PET-CT myocardial perfusion imaging (MPI) with Regadenoson (pharmacologic stress agent) and was incidentally found to have a focus of uptake in the outer right breast with an enlarged right axillary lymph node (Fig. 1). No prior mammograms were available and it is unknown whether the patient was undergoing routine screening mammography at an outside facility. The patient had no personal or family history of breast cancer. She underwent diagnostic mammogram, which did not reveal any definite correlate for the activity on PET (Fig. 2). Ultrasound was performed on the same day, which revealed a mass located adjacent to the implant capsule (Fig. 3). She underwent ultrasound-guided biopsy and postclip mammography which revealed a diagnosis of atypical ductal hyperplasia with a background of fibrous stroma. The patient underwent subsequent seed-localized excisional biopsy as is the protocol for atypical ductal hyperplasia. Final pathology at excision demonstrated ductal carcinoma in situ (Grade 2), cribriform type. Subsequent surgery revealed ductal carcinoma in situ. She underwent re-excision to negative margins 2 weeks after the first surgery.

## Discussion

Incidental detection of breast cancer on cardiac imaging studies has been extensively described in the literature. As Tc-99m sestamibi replaced more traditional radiopharmaceuticals for cardiac stress testing in the early 1990s, it was quickly discovered that sestamibi was taken up by breast cancers [4]. The use of sestamibi for detection of breast cancer led to the development of molecular breast imaging, an adjunct screening tool that is still in use today.

Over the last 2 decades, PET imaging has further refined myocardial perfusion imaging [5]. The advantages of myocardial PET imaging include higher myocardial resolution, detection of microvascular disease, and combined regional and global myocardial blood flow [6]. These characteristics allow for a better diagnostic and therapeutic approach for patients with cardiovascular disease [7]. While Tc-99m Sestamibi SPECT (single photon emission computed tomography) remains the most widely available and utilized form of radiotracer-based evaluation for ischemia in patients with suspected coronary disease, it is often subject to significant limitations that may affect its diagnostic accuracy [7]. Some of these include soft tissue attenuation artifacts, balanced myocardial ischemia (in which significant disease in all myocardial vessels results in a homogeneous myocardial picture which fails to demonstrate perfusion defects), as well as significant subdiaphragmatic [7]. PET-CT overcomes these challenges, results in less radiation exposure for patients and provides clinicians with regional myocardial blood flow estimates [7]. Additionally, with PET-CT stress tests, images often include a low-resolution CT-image correlate as an anatomical reference. These CT-image correlates, referred to as the transmission image, affords the reading physician the ability to evaluate extracardiac tissues and detect incidentalomas that may be highlighted by the presence of radiotracer uptake, such as breast cancer [8,9].

Breast ductal neoplasms exist on a spectrum, from benign breast disease to invasive ductal cancer (IDC). This spectrum includes atypical ductal hyperplasia (ADH), characterized by an abnormal overgrowth of cells lining the milk ducts, which increases an individual's risk of developing breast cancer later in life. While ADH increases the absolute risk of breast cancer by 30%, not all ADHs progress to invasive disease, which is why it is considered a direct, but not obligate precursor to disease [10]. ADH resembles a low-grade DCIS, with similar atypical nuclear and architectural features. ADH is often identified on core needle biopsy. If evidence of DCIS is found on subsequent surgical excisional biopsy, the lesion is upgraded [10], as was the case in our patient. Given the high upgrade rate, many clinicians counsel patients to proceed with surgical excisional biopsy as an appropriate next step.

## Conclusion

With the increasing sophistication of myocardial perfusion imaging, extracardial findings of significance, such as breast cancer, are important to recognize. In our case, recognition of abnormal uptake of radiotracer in the breast resulted in a prompt diagnosis, treatment, and favorable prognosis.

## Patient consent

The patient provided written consent for anonymized images and history to be included in publications.
